# Effectiveness of the Metacognitive Training–Silver Program in Reducing Depressive Symptoms and Enhancing Metacognitive Functioning in Elderly Adults with Depression: A Randomized Controlled Trial

**DOI:** 10.1192/j.eurpsy.2026.10666

**Published:** 2026-07-31

**Authors:** M. Murat Mehmed Ali, S. Buzlu, L. Guedes De Pinho

**Affiliations:** 1 Institute of Graduate Studies; 2 Mental Health and Psychiatric Nursing Department, Istanbul University - Cerrahpaşa, İstanbul, Türkiye; 3 Escola Superior de Saúde, Instituto Politécnico de Viana do Castelo, Viana do Castelo, Portugal

## Abstract

**Introduction:**

Depression in older adults significantly reduces quality of life and is commonly associated with dysfunctional attitudes and negative perceptions of aging. While pharmacotherapy remains the dominant treatment modality, its limitations in fully addressing underlying cognitive mechanisms have underscored the need for innovative and sustainable psychosocial interventions. The Metacognitive Training–Silver (MCT-Silver) program is designed specifically to enhance cognition and metacognition in the elderly.

**Objectives:**

This study aimed to evaluate the effectiveness of the MCT-Silver program in improving depressive symptoms, dysfunctional attitudes, metacognitive beliefs, and perceptions of aging in older adults diagnosed with depression.

**Methods:**

This randomized controlled experimental study employed a pretest-posttest design with 3-month follow-up. A total of 50 elderly individuals (≥60 years) with a clinical diagnosis of depression were randomized into intervention (n=24) and control (n=26) groups. The intervention group received an 8-week MCT-Silver program, while the control group received routine care. Data were collected at three time points using validated tools: Beck Depression Inventory, Dysfunctional Attitude Scale, Metacognition Questionnaire-30, and the Attitudes to Aging Questionnaire. Statistical analysis included Pearson Chi-Square, independent samples t-test, Mann–Whitney U, and non-parametric repeated measures ANOVA.

**Results:**

Participants’ mean ages were 67.50 ± 5.28 (intervention) and 68.88 ± 5.04 (control), with no baseline differences. Compared to the control group, the intervention group demonstrated statistically significant reductions in depressive symptoms (post p<0.001; 3-month follow-up p<0.001), dysfunctional attitudes (post p=0.002; follow-up p<0.001), and maladaptive metacognitive beliefs (post and follow-up p<0.001). Additionally, significant improvements were observed in attitudes toward aging at both post-intervention and follow-up assessments (p<0.001). No adverse events were reported.

**Conclusions:**

Findings indicate that the MCT-Silver program is a promising and feasible intervention for older adults with depression. It not only reduces depressive symptoms but also modifies dysfunctional cognition and enhances metacognitive capacity, contributing to a more adaptive and positive perception of aging. Incorporating this program into psychiatric nursing practice may contribute to enhanced geriatric mental health outcomes and support holistic, nurse-led interventions in late-life depression management.

**Disclosure of Interest:**

None Declared

